# The role and scope of practice of forensic podiatry

**DOI:** 10.1186/1757-1146-3-S1-O26

**Published:** 2010-12-20

**Authors:** Wesley Vernon, Jeremy Walker, Sarah Reel, Haydn Kelly, Brian Brodie, John DiMaggio, Michael Nirenberg, Norman Gunn

**Affiliations:** 1 Sheffield PCT, Sheffield, UK

## Objectives and relationship to conference themes

The presentation will provide an overview of the role and scope of practise of forensic podiatry. This is a new and innovative area of practice, often involving footwear and as such, the presentation fits in with these conference themes.

## Content

A document has been prepared by an international committee on forensic podiatry defining the role and scope of practise of this new discipline for the first time. This document will be presented and explained. The presentation will explain how the role and scope of forensic podiatry came to be defined and will cover the definition of forensic podiatry, the level of education expected and the tasks performed by forensic podiatrists. In view of the potential for confusion where forensic investigations involve multiple disciplines, the presentation will also consider areas in which forensic podiatrists would not become involved.

## Relevance/impact

As a very new area of practise, the paper will provide a timely opportunity to enhance delegates understanding of forensic podiatry and how this fits in with the wider forensic world.

## Participant outcomes

To understand how to approach this field. To help delegates understand how to avoid the pitfalls and dangers of working in forensic podiatry.

